# Development and validation of the CAREGIVERS questionnaire: multi-assessing the impact of juvenile idiopathic arthritis on caregivers

**DOI:** 10.1186/s12969-020-0400-z

**Published:** 2020-01-14

**Authors:** Marcia Daniela Torres-Made, Ingris Peláez-Ballestas, Fernando García-Rodríguez, Ana Victoria Villarreal-Treviño, Brenda de Jesús Fortuna-Reyna, Manuel Enrique de la O-Cavazos, Nadina Eugenia Rubio-Pérez

**Affiliations:** 1 Department of Pediatrics, Universidad Autónoma de Nuevo León, Hospital Universitario “Dr. José E. González”, Monterrey, Mexico; 2 Rheumatology Unit, Hospital General de México “Dr. Eduardo Liceaga”, Mexico City, Mexico

**Keywords:** Juvenile idiopathic arthritis, Caregiver, Questionnaire, Burden, Multi-assessment, Pediatric rheumatic diseases

## Abstract

**Background:**

The primary caregiver is an important person in the life of patients with JIA. Their reactions depend on social, emotional and economic factors that affect the therapeutic alliance. Some generic instruments have been used to evaluate burden, anxiety, or quality of life of caregivers. This study aims to develop a specific instrument to measure the psychosocial and economic impacts on primary caregivers of patients with JIA.

**Methodology:**

This is a mixed methods research, that includes qualitative and quantitative data, and was carried out in two phases. First phase: a pragmatic qualitative study (questionnaire construction) was conducted in two parts, a non-systematic literature review followed by interviews with primary caregivers. Second phase: a cross-sectional study (questionnaire validation) to complete validation and estimate Cronbach’s alphas based on tetrachoric correlation coefficients, correlation matrix and Cohen’s kappa coefficient test.

**Results:**

There were 38 articles found related to the experience of caregivers. 15 primary caregivers were interviewed (female 93%, median age 45 years). Thematic analysis identified 9 important topics from the perspective of participants (economic impact, coping, family roles, impact of diagnosis, mental health, couple/mate relationships, impact at work, religion, and knowledge of the disease). These topics were combined to create the interview questionnaire (56 items). Later, it was modified to 62 items that were divided into five dimensions: impact of the disease (psychosocial, economic, family, and relationships), knowledge of the disease, alternative medicine, future, and religion.

The interview questionnaire was applied to 32 primary caregivers (female 93%, median age 37 years), results identify depression on 29 (90%), 18 (56%) feel sadness at diagnosis, 20 (63%) mentioned that JIA has influenced in their financial situation, 23 (72%) feel anxiety about the future, and 11 (37%) considered that their family relationships have changed.

Statistical analysis identified inconsistencies during convergent and divergent validity of the construct. Consequently, 11 items were eliminated, 3 relocated, 6 modified, and 39 compacted obtaining the “Impact of Pediatric Rheumatic Diseases on Caregivers Multi-assessment Questionnaire” (CAREGIVERS questionnaire). This final version resulted on an eight-dimension (28 items) instrument.

**Conclusions:**

The CAREGIVERS questionnaire captures perspectives of both the participants and clinicians. It will be helpful to measure the impact of the disease and thus, to improve the quality of care of children with JIA and their families.

## Background

Pediatric rheumatic diseases (PRD) constitute a heterogeneous set of disorders linked to abnormalities in the functioning of the immune system. The main characteristic of this type of pathology is inflammation of the connective tissue, especially the joints, blood vessels, and skin [1].

The most common PRD is juvenile idiopathic arthritis (JIA) that is a group of chronic diseases in children and adolescents characterized by joint pain and inflammation, with limited range of movement, functional disability, and structural damage over the years [2]. JIA can influence psychosocial, economic, physical and educational development, affecting the quality of the life of children and their families [3, 4].

The primary caregiver is an important person in the life of patients with JIA, since it is the main support to carry out medical instructions to control the disease [5–7]. The reactions of the caregiver to JIA depend on many factors such as experience in crisis situations and medical problems, socio-economic status, local culture, knowledge of the disease, quality of health services and support networks. This may affect the therapeutic alliance and adherence of the patient [4, 5, 8–10].

Some instruments have been created to measure burden, anxiety, depression or quality of life of caregivers, however, those have not been designed for JIA [11–13]. None of these instruments integrates social, emotional, economic, and physical issues in a single questionnaire, which are frequently affected areas in caregivers [6, 7, 14]. Therefore, it is necessary to increase research that contributes to the understanding of the burden of JIA on caregivers and their influence on the outcomes. We hypothesize that an instrument specifically designed for those taking care of JIA patients will be better for capture their needs and interests. In addition, it will make the evaluation more efficient by integrating several dimensions into a single questionnaire.

The aim of this study is to develop and to validate a specific instrument that measures the psychosocial and economic impact on primary caregivers of children and adolescents with JIA.

## Methodology

### Study design

**Fig. 1:**
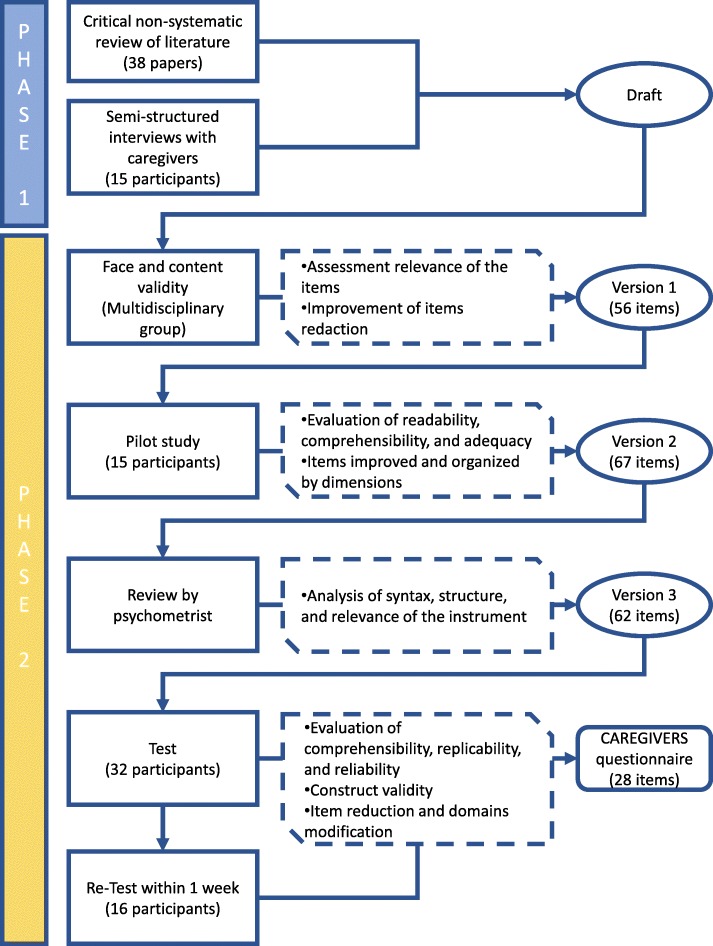
Overview of the questionnaire construction and validation process

This is a mixed methods research study, that includes qualitative and quantitative data, and was carried out in two phases [15–17]. First phase: a pragmatic qualitative study (questionnaire construction) was conducted in two parts, a non-systematic literature review followed by interviews with primary caregivers. Second phase: a cross-sectional study (questionnaire validation) validity were completed (Fig. 1).

The literature review, designing of the items, and face validity were conducted by a multidisciplinary group of professionals. It was formed by 3 pediatric rheumatologists (certified by Mexican Rheumatology Board and with at least 3 years of clinical practice), one fellow in pediatric rheumatology, one psychologist with experience in chronic diseases, and a methodologist with expertise in the development of questionnaires.

This research received approval by local Ethics Committee (PE17–00012), and written informed consents were collected from each participant.

### Participants

Between April 2017 and April 2018, we prospectively included primary caregivers, of both genders, of patients with JIA (according to International League of Associations for Rheumatology classification criteria [18]) who attend the Pediatric Rheumatology Clinic of Hospital Universitario “Dr. José Eleuterio González” or researchers (NER-P and FG-R) private practice. All the participants had to be a close family member of the patient (parent, older sibling, grandparent/grandmother, or aunt/uncle) and did not receive any kind of payment for their care. We excluded caregivers of patients that were admitted to the hospital 4 weeks prior, caregivers who were diagnosed with a chronic disease, caregivers that care for more than one patient with a chronic disease and caregivers who refused to participate.

### Sample

A convenience sampling was applied, basing sample size on the quality criteria proposed by Terwee et al. [19], who suggested that 30 patients were needed to construct validation. We also included 15 additional participants to conduct the first phase interviews and another 15 in a pilot study of the questionnaire’s first version. The semi-structured interviews provided thematic saturation [20] regarding the caregivers’ experience and their impact taking care of JIA patients.

Participants were selected to obtain a heterogeneous sample; these include different ages, patient’s JIA category, education, and social environment.

### Phase 1. Construction of the questionnaire

#### Non-systematic literature review

Items for the questionnaire were first based on a non-systematic review of the literature using the following databases: MEDLINE, ProQuest and Scopus. We looked for terms related to caregiving and chronic childhood illnesses (not only PRD) to identify the more relevant issues associated with the burden of caregivers (Additional file 1 and Additional file 2). Then, papers were individually analyzed by the multidisciplinary group and discussed to generate a list of items that must be considered for the instrument.

#### Semi-structured interviews

A pragmatic qualitative study [21–23] with face-to-face interviews was conducted with 15 participants with the objective of identifying which areas of their life were impacted after the illness was diagnosed. The caregivers that participated in this phase were not included in the validation stage.

The interviewer asked the caregiver to participate, if agreed, they were invited to a private room for the interview to be carried out. The interview was recorded for further analysis. No incentives were offered for their participation.

The participants followed a semi-structured interview specifically designed for the purpose of this study. A guide for the interview was constructed by the multidisciplinary group based on the results of the literature review and their own experience. Topics investigated were mood, emotional problems, suffering of the disease, family issues, difficulties in obtaining medical attention, financial problems, religion, beliefs and supporting networks. During the interview, they were asked to explain their experiences and emotions as care holders, also look at each topic and consider whether those referred to relevant aspects of the caregiver’s burden. They were also asked if they considered that any items were missing that could be beneficial for the questionnaire. The participants were not manipulated in any way by the interviewer to obtain certain answers.

A thematic analysis was performed of the recorded interviews transcriptions. Important topics were identified from the perspective of the participants; then, analysis and interpretation were completed by the multidisciplinary group during several meetings [22, 24].

We integrated information that was collected from the literature review with the results of the thematic analysis to construct the first draft of the instrument. The questions and response categories were based on phrases used by the participants during the interviews to avoid technical terms.

### Phase 2. Validation of the questionnaire

During face and content validity, the multidisciplinary group met to assess the relevance of the items drafted to create the first version of the questionnaire. This was tested in a pilot study with 15 participants. After the group analyzed the results, it was decided that questions should be organized by dimensions (mental health, socioeconomic impact, supporting networks, and new topics) and minor revisions were made to obtain the second version of the questionnaire. This version was reviewed by a psychometrist to obtain a third version (Fig. 1).

The third version of the questionnaire was applied to 32 participants. Quality of life (Spanish Version of EuroQol instrument: EQ-5D-3 L, 14 items), coping (Reyes-Lagunes and Góngora-Coronado, UNAM 1996, adapted dimensions “When I have problems with my children’s health” and “When I have problems in my life”, 18 items each), socioeconomic impact (“Determinación del Impacto Económico en Enfermedades Reumáticas” instrument, short version, 26 items), and depression (Spanish Version Beck’s Depression Inventory, 21 items) questionnaires were also applied to participants to demonstrate the validity of the construct.

The questionnaire was applied to 50% of the participants 7 days after the first test to evaluate the reliability of the instrument (test-retest).

After validation results were analyzed by the multidisciplinary group, modifications were made to create the final version of the questionnaire.

### Qualitative and quantitative analysis

The analysis was performed according to the phase. We performed qualitative analysis using ATLAS/ti software, where relevant topics were identified by transcription of the interviews. After construction of the first version of the questionnaire, face validity was made as described above.

The quantitative analysis was carried out after data was collected and coded from the questionnaires. Central tendency and dispersion statistics were estimated for demographic characteristics of participant.

The assessment of the internal consistency was done by estimating the Cronbach’s alphas for each dimension of the questionnaire based on tetrachoric correlation coefficients of the items. Alphas equal to or greater than 0.40 (*p* < .05) were considered acceptable [25, 26].

A correlation matrix of variables of each dimension were carried out to look for redundancies and reduce the questionnaire. For pairs of variables with correlation coefficients higher than 0.70, we selected the one that explained the greatest variation.

External consistency or reliability was estimated with the test-retest. It was evaluated using Cohen’s kappa coefficient test, comparing the scores obtained between the first and the second evaluation in 50% of the participants to whom the same instrument was applied twice within a 7 days interval. All statistical analyses were carried out using Stata V. 11.

## Results

### Phase 1. Construction of the questionnaire

During the literature review, 38 articles were found related to the experience of caregivers on pediatric patients with chronic diseases (Additional file 1 and Additional file 2).

The interviews (10 performed by a medical anthropologist and 5 by a senior fellow on Pediatric Rheumatology) lasted 15 to 20 min each. Participants were mostly female (93%), median age was 45 years (ranged 28 to 60) and reporting 11.7 years of schooling (ranged 0 to 20). After the thematic analysis, we identified important topics from the perspective of participants (Table 1). We used them to create a draft of the questionnaire.

**Table 1:** Results from thematic analysis of interviews transcriptions

Topics	Interview transcription
Economic impact	• *“Where are we going to get us [so much money]? Because [my husband] works in a transport truck and I work at home [handmade] manual labor. We have four children, everyone goes to school, and we pay for electricity, water, telephone.” (Female, 31 years old)* • *“Yes, worrying because right now we have not reached the limit that we have to buy [medication], but the moment they say ‘you know what? You already have to buy the medicine’, I do not know how we are going to do it. We have to see how we do it: sell, do, get, borrow, pawn. I do not know, or work more. The problem is already there latent.” (Female, 28 years old)*
Coping	• *“Do not leave her alone, support her in everything, and ask God to help us to move forward and face the disease.” (Female, 34 years old)*
Family care roles	• *“I used to take decisions about care of him [patient] regularly because he [father] worked at a certain time of day and I understood it that way, I said we cannot walk, I carried him [patient] and I have to do it, when it was maybe a stronger decision, then I call to my husband” (Female, 28 years old)*
Impact of diagnosis	• *“When they confirmed the diagnosis... Ah, I felt calmed because I said well, we’ve been playing like little balls from one place to another, and when they find the problem, it feels a relief because it says well, they know how to control it, I know that there is no cure, but yes, one feels bad definitely.” (Female, 30 years old)*
Mental Health	• *“I was very depressed to see her with this illness, and to think that she would be like this for the rest of her life.” (Female, 41 years old)*
Couple/mate relationships	• *“I always felt very lonely with her, because her father did not pay attention to her. He never supported us, he didn’t believe in the disease, he said that she wasn’t sick.” (Female, 38 years old)*
Impact at work	• *“I asked permission [to leave work] if there was any question during the morning, but no, I did go ahead, because he [patient] transmitted [to me] his strength, because he has always been very strong, my son.” (Female, 31 years old)*
Religion	• *“The religion definitely has helped me. Something that I know, is that God is love.” (Male, 30 years old)*
Knowledge of the disease	• *“Not much, I know what he [doctor] has told me, I have read about the disease on the Internet, sometimes I have not wanted to go too far because one is afraid of the unknown; yes, then, I prefer, sometimes, not to search, [not to] find.” (Female, 37 years old)*

### **Phase 2. Validation of the questionnaire** (Fig. 1)

During face validity, the draft was modified to create Version 1 of the instrument, which included 56 items, 50 with categorical answers and 6 open-ended questions.

The pilot study performed with 15 participants (80% female, median age 39 [ranged 26 to 63] years) shown confusion in 3 items, that were restructured, and 6 were eliminated upon finding them reiterative. The need for questions that looked deeper into caregivers’ relationships (e.g. mates and patients), future expectative, alternative medicine, social networks, and religion were also identified. Consequently, items were created and added to the instrument to complete Version 2. The multidisciplinary group considered it appropriate to divide this version into five dimensions for further analysis: impact of the disease (psychosocial, economic, family, and relationships), knowledge of the disease, alternative medicine, future, and religion.

After the psychometrist reviewed Version 2, 6 items were confusing and therefore modified, 5 were eliminated for being redundant. Items were organized in the same five dimensions to obtained Version 3. Evolution of questionnaire composition are shown in Table 2.

**Table 2:** Evolution on numbers of items and domains during questionnaire construction

Dimensions	Number of items		
	Version 1	Version 2	Version 3
I. Impact of the disease			
1.Psychosocial impact	11	13	13
2.Economic impact	12	13	12
3.Family impact	5	5	4
4.Relationship impact	0	2	2
II. Knowledge of the disease	28	28	26
III. Alternative medicine	0	2	1
IV. Future	0	2	2
V. Religion	0	2	2
Total	56	67	62

Version 3 and the other 4 tools were applied to 32 caregivers for the validity analysis. Characteristics of patients and participants are presented in Table 3. Coping strategies identified were mainly emotional-negative (31 participants, 97%).

**Table 3:** Characteristics of the patients and caregivers participating in the construct validation of the version 3 (*n* = 32)

		Patients	Caregivers
Age in years, median (IQR)		13 (10–18)	37 (33–46)
Female (%)		25 (78)	30 (93)
Public medical access (%)		25 (78)	NA
Time to the center in hours, median (IQR)		1 (0.5–1.5)	NA
JIA category^a^ (%)	Oligoarticular	2 (6)	NA
	Polyarticular^b^	24 (75)	
	Systemic	3 (9.5)	
	Psoriatic	0 (0)	
	Enthesitis related	3 (9.5)	
	Undifferentiated	0 (0)	
History of uveitis (%)		0 (0)	NA
History of MAS (%)		1 (3)	NA
History of hospitalization (%)		4 (12.5)	NA
Presence of any disability (%)		4 (12.5)	NA
Active disease at enrollment (%)		9 (28)	NA
Treatment (%)	NSAIDs	21 (65)	NA
	Synthetic DMARDs	21 (65)	
	Steroids	2 (6)	
	Biologic DMARDs	10 (31)	
Depressive symptoms (%)^c^		NA	29 (90%)
VAS on EQ-5D-3 L, median (IQR)		NA	82 (75–91)
Had a remunerated job (%)		NA	19 (59)
Medium socioeconomic status or above (%)		NA	22 (69)
Education level: Highschool or above (%)		NA	18 (56)

*IQR* Interquartile range, *MAS* Macrophage Activation Syndrome, *NSAIDs* Nonsteroidal anti-inflammatory drugs, *DMARDs* Disease-modifying antirheumatic drugs, *VAS* Visual analogue scale, *EQ-5D-3 L* Spanish Version of EuroQol instrument, *NA* Not applicable

^a^According to International League of Associations for Rheumatology classification criteria

^b^Both rheumatoid factor positive and negative JIA

^c^According to Spanish Version Beck’s Depression Inventory (21 items)

Data from our questionnaire revealed that 18 (56%) participants felt sadness when the diagnosis was confirmed, but in 27 (84%) of them change through time, and currently 14 (44%) feel relief. Twenty (63%) participants mentioned that JIA has influenced their financial situation, 23 (72%) felt anxiety about the future of their children, and 11 (37%) considered that their family relationships have changed since the diagnosis. Bullying was reported from 10 (31%), nine has used social networks to interact with people in the same situation, and 29 (91%) have an established religion. All the participants knew the name of the disease and treatment of their patients. The questionnaire was well tolerated by the participants, taking 18 min (IQR 16 to 21) to answer and only 0.2% of missing data.

Statistical analysis identified inconsistencies during convergent and divergent validity of the construct, carried out with correlation matrices (Additional file 3). Cronbach’s alphas derived from internal consistency assessment of the third version are shown on Table 4. The test-retest reliability evaluation found disagreements in 6 questions.

**Table 4:** Results from internal consistency analysis of the third version of the questionnaire

Dimensions	Cronbach’s alpha
I. Impact of the disease	
1.Psychosocial impact	0.42
2.Economic impact	0.69
3.Family impact	0.23
4.Relationship impact	0.20
II. Knowledge of the disease	0.20
III. Alternative medicine	0.54
IV. Future	0.31
V. Religion	0.004

Based on the qualitative and quantitative validation analysis, the third version of the questionnaire was adjusted: 11 items were eliminated, 3 relocated, 6 modified, and 39 compacted in 14 items; domains were also modified. With this, final version of the “Impact of Pediatric Rheumatic Diseases on Caregivers Multi-assessment Questionnaire” (CAREGIVERS questionnaire) resulted in an eight-dimension (28 items) instrument (Additional file 4).

## DISCUSION

The CAREGIVERS questionnaire shown a good understanding among participants, reliability, and consistency to measure the psychosocial and economic impact on caregivers of patients with JIA. It represents the first step to establish a program of research into demands of caregivers and becomes an effort to improve their conditions. The instrument content is supported by the literature review, opinions from a multidisciplinary group of professionals and what the caregivers expressed during the interviews. Besides, qualitative and quantitative evaluation derived in critical analysis that assured the questionnaire in a comprehensive, relevant and practical way to administer.

The primary caregiver is the adult that lives with and is the most active in caring for a child [11, 27]. In children with PRD, the caregiver’s responsibility increases because of complex medical therapies and long disease activity periods, trying to combine those issues with the patient’s rise and family functioning [13, 28]. Previously, positive and negative impacts have been described on caregivers [6, 7, 13, 29–31], but little is known about how diseases affect individual needs, social interactions, family economy, and emotions. Therefore, it is imperative that we study the impact of illness-related to caregiving on this population.

Results show a stable instrument, even though problems in internal consistency were identified, resulting in low alpha scores in “Knowledge of the disease” and “Religion” dimensions. This situation was due to an initial excessively large construction (with many elements) in “Knowledge of the disease” dimension. We also detected high variability in answers (due to the inclusion of open-ended questions) and that the dimension had not influenced on caregivers. Even more, we considered that the goal of the instrument was to evaluate the impact of the disease and not in the knowledge, this dimension was eliminated. In the “Religion” dimension, the two original items show a narrow and opposite spectrum of responses, thus were compacted in one item and expanded the answer options.

Multiple areas have been described as affected in caregivers of patients with JIA, some of those appeared during the descriptive analysis of the population. In other studies, changes were reported in relationships and role distribution in families, emotional distress experiences, appraisal of illness uncertainty, elevated out-of-pocket costs and limited time for other activities [10, 13, 14, 28, 32–34]. Results also show that caregivers often avoid to reveal their emotions to other family members [13, 14, 35]. This was similar to the attitudes found in our population, specially related with the lack of supporting networks. All these topics are addressed in the proposed instrument.

Previous research has focused more on caregiver characteristics and patient’s well-being but little has been investigated about family composition, relationships, social and support networks for caregivers. Moreover, the evaluations were made with a wide variety of instruments [7, 12, 28–30, 33, 34, 36] or through interviews and qualitative analysis [6, 7, 12–14, 31, 32, 34]. Approaching those issues in a multi-assessment manner is the most relevant contribution of the CAREGIVERS questionnaire, which could help to identify patterns of caregiver response and their relationship to patients’ outcome.

There is a lack of information about programs based on emotional health of the caregiver or specific data on the relationship of caregiver problems and final outcome of JIA [35–37]. However, interventions and social supporting programs to patient and their caregivers results in development of positive coping mechanisms, improving stress management and decreasing anxiety and depression [1, 8, 37]. Studies on this field will be benefited with this instrument because it could provide a lot of important information with a simple evaluation.

The questionnaire is proposed to be used as a multidimension screening instrument for burden of caregivers. It allows to examine the impact of JIA on several areas of the caregiver’s life, looking for specific concerns and needs in which treatment could be required.

We found some limitations in our study, the first was the relatively small sample size. JIA is a disease with a low prevalence, therefore the sample requested by clinimetric and psychometric studies is smaller, and it is difficult to concentrate a large number of patients with this pathology. The second limitation is that the validation process was not completed since the sensitivity to change and external validity was not accomplished. This will be done in a multicentric study with a larger sample size that is currently conducted by the authors.

Designing a multidimensional questionnaire from the perspective of those who bear the burden of the disease is a complex process that requires the participation of caregivers and clinical and psychological providers. The CAREGIVERS questionnaire accomplished the balance between those aspects; it will be helpful to measure the impact of the disease and therefore, to improve the quality of care of children with JIA and their families.

Finally, the objective of this work was to focus on the caregivers for JIA patients, because it is the most frequent PRD [2] however, being so valuable, we propose to consider further studies to evaluate its application in other diseases.

## Conclusion

The CAREGIVERS questionnaire shown a good understanding among participants, reliability and consistency to measure the psychosocial and economic impact on primary caregivers of patients with JIA. It is necessary to complete the external validation of the instrument in further studies.

## Supplementary information

**Additional file 1. ** Description of the non-systematical literature review**.**

**Additional file 2.** Supplementary figure: Overview of search results and review.

**Additional file 3.** Correlation matrix resulted from construct analysis.

**Additional file 4.** CAREGIVERS questionnaire: Impact of Pediatric Rheumatic Diseases on Caregivers Multi-Assessment Questionnaire.

## Data Availability

The datasets used and/or analyzed during the current study are available from the corresponding author with a reasonable request.

## Abbreviations

CAREGIVERS questionnaire: Impact of Pediatric Rheumatic Diseases on Caregivers Multi-assessment Questionnaire
EQ-5D-3 L: Spanish Version of EuroQol instrument
IQR: Interquartile range
JIA: Juvenile Idiopathic Arthritis
PRD: Pediatric rheumatic diseases
UNAM: Universidad Nacional Autónoma de México
