# Supplementary material for: Development and validation of the CAREGIVERS questionnaire: multi-assessing the impact of juvenile idiopathic arthritis on caregivers
Source: Pediatr Rheumatol Online J. 2020 Jan 14;18:3. doi: 10.1186/s12969-020-0400-z (PMC6961380; doi:10.1186/s12969-020-0400-z)
Supplement: Supplementary file 1 — Additional file 1. Description of the non-systematical literature review. [file 12969_2020_400_MOESM1_ESM.docx]

**Supplementary file 1. Description of the non-systematical literature review.**

Supplementary figure. Overview of search results and review (Supplementary File 2).

Supplementary table. Description of articles included to create the instrument.

| Author | Year | Methodology | Data collection | Participants | Main results |
| --- | --- | --- | --- | --- | --- |
| Bouma and Schweitzer([1](#_ENREF_1)) | 1990 | Cross-sectional study. | Questionnaire on Resources and Stress Short Form. | 72 mothers of children:   - 24 cystic fibrosis. - 24 autism. - 24 without a physical or psychological disorder. | Different patterns of stressful response.  Autism contributes significantly more to family stress than cystic fibrosis. |
| Wells et al.([2](#_ENREF_2)) | 2002 | Instrument development and initial testing. | Literature review of related studies and meetings of pediatric  oncology nurses. | An expert review panel of pediatric oncology advanced practice  Nurses.  158 caregivers of children with cancer. | Items needs to be considered or improved:   - Planning activities for the family and the ill child. - Improve reporting to the treatment team. - Meeting the emotional needs of the ill child, spouse, other children, extended family, and own needs. - Managing of painful events. - Getting information about child’s illness. - Communicating about child’s illness. - Managing finances. - Maintaining child’s comfort. - Managing side effects. - Managing siblings’ illnesses. - Attending medical appointments. - Managing of additional household tasks. - Managing unexpected events. - Obtaining childcare for siblings. |
| Pelaez-Ballestas et al.([3](#_ENREF_3)) | 2006 | Qualitative study. | Recordings and verbatim transcripts of in-depth interviews. | 10 families:   - 16 parents - 6 children | 'Pilgrimage' as main concept:   - Parents: reflect the religious reference to the trajectory of pain, faith and hope. - Children: conformed by immediate concepts. - Influenced by JIA subtype. |
| Ryan et al.([4](#_ENREF_4)) | 2013 | Cross-sectional study. | Measures of caregiver demand, parental distress, and illness attitudes. | 70 patients with JIA and their parents. | Hierarchical regression relationships between:   - Caregiver demand and parental distress. - Caregiver demand and parent illness attitudes. - Parent illness attitudes and parental distress. |
| Mawani et al.([5](#_ENREF_5)) | 2013 | Cross-sectional study. | Clinical records and the Caregiver Reaction Assessment (CRA). | Primary caregiver of 47 JIA patients. | Affected dimensions on CRA:   - Self-esteem - Financial problems (main) - Health problems - Disrupted schedule (main) - Familial support.   Positive feelings:   - Satisfaction - Gratitude |
| Santer et al.([6](#_ENREF_6)) | 2014 | Systematic review with thematic synthesis of qualitative studies. | Papers contained qualitative data of parents and caregivers of children with long-term conditions. | 19 documents:   - 17 studies (423 family groups from 5 countries) | Factor influencing adherence:   - Beliefs about disease and treatment. - Difficult on treatment. - Children resistance to treatment. - Family relationships. - Look for a normal life. |
| Knafl et al.([7](#_ENREF_7)) | 2015 | Mixed-methods synthesis review and meta-analysis. | Research reports addressing the intersection of family life and JIA. | 29 studies. | Meta-analysis results:   - Moderately strong positive relationship between parent and child psychological functioning. - Medium negative relationship between family conflict and child psychosocial well-being. - Medium negative relationship between parental depression and child physical functioning.   Thematic analysis:   - Caregiver role different from parent role (conflicts being both). - Caregiving responsibilities related to severity of disease. - Pain management is a main issue. |
| McDonagh et al.([8](#_ENREF_8)) | 2016 | Qualitative study. | Private on-line blog | 36 patients with JIA and 6 parents. | Opinions and feelings on:   - Early and delayed diagnosis - Identity building effects - Impact on adolescence - Looking for a positive effect - Facing future - Medication effect on life - Adverse events impact |
| Gómez-Ramírez et al.([9](#_ENREF_9)) | 2016 | Qualitative study. | Recordings and verbatim transcripts of focus groups sessions.  Written reports of reciprocal interviews. | 23 parents of JIA patients:   - 15 experienced parents - 8 novice parents | During diagnosis workout:   - Feelings of confusion, anxiety, frustration, fear, and anger.   After diagnosis:   - Shock, surprise, fear, hope, and regrets.   During a controlled disease:   - Fear, hope, gratitude, relief, fatigue, anxiety, and frustration.   During flares:   - Frustration, anger, sympathy, and admiration.   During course of the disease:   - Anxiety and defenseless. |
| Chaney et al.([10](#_ENREF_10)) | 2016 | Cross-sectional study. | Clinical data provided by rheumatologist.  Instruments:   - Child Depression Inventory - Parent Perceptions of Uncertainty Scale - Care for My Child with Rheumatic Disease Scale   Brief Symptom Inventory. | 82 children with rheumatic diseases (JIA, JDM, JSLE and others) and their parents. | Associations:   - Illness uncertainty and caregiver demand. - Caregiver demand and both parent distress and child depressive symptoms. |
| Chausset et al.([11](#_ENREF_11)) | 2016 | Mixed methods study. | Semi-structured interviews of families. | 11 families of patients with JIA. | Thematic analysis:   - The path of parents was characterized by doubt while the disease tended to take center stage. - Doubt was generated. - Social support and parent associations occupied an ambiguous position between help and stigmatization. - Feelings of shock, worry, uncertainty, guilt, hope, and relief during disease course. |
| Yuwen et al.([12](#_ENREF_12)) | 2017 | Qualitative study. | Single-occasion in-depth interviews. | 9 parents   - 8 mothers   1 father | Inductive content analysis:   - Not knowing: overwhelmed about information. - Trying to reach out in the dark: Nobody understands them and feel lonely. - Feeling my child's pain. - Working out the kinks to stay on top to manage: Tried to stay on top of the child's illness and treatment, even when they felt drained physically and emotionally. - Feeling drained by the whole process. - Being hard on the entire household: JIA affects the entire family. |

JIA: Juvenile Idiopathic Arthritis. JDM: Juvenile Dermatomyositis. JSLE: Juvenile Systemic Lupus Erythematosus.

List of articles included.

1. Bouma R, Schweitzer R. The impact of chronic childhood illness on family stress: a comparison between autism and cystic fibrosis. J Clin Psychol. 1990 Nov;46(6):722-30.

2. Wells DK, James K, Stewart JL, Moore IM, Kelly KP, Moore B, et al. The care of my child with cancer: a new instrument to measure caregiving demand in parents of children with cancer. J Pediatr Nurs. 2002 Jun;17(3):201-10.

3. Pelaez-Ballestas I, Romero-Mendoza M, Ramos-Lira L, Caballero R, Hernandez-Garduno A, Burgos-Vargas R. Illness trajectories in Mexican children with juvenile idiopathic arthritis and their parents. Rheumatology (Oxford). 2006 Nov;45(11):1399-403.

4. Ryan JL, Mullins LL, Ramsey RR, Bonner MS, Jarvis JN, Gillaspy SR, et al. Caregiver demand and parent distress in juvenile rheumatic disease: the mediating effect of parent attitude toward illness. J Clin Psychol Med Settings. 2013 Sep;20(3):351-60.

5. Mawani N, Amine B, Rostom S, El Badri D, Ezzahri M, Moussa F, et al. Moroccan parents caring for children with juvenile idiopathic arthritis: positive and negative aspects of their experiences. Pediatr Rheumatol Online J. 2013 Oct 20;11(1):39.

6. Santer M, Ring N, Yardley L, Geraghty AW, Wyke S. Treatment non-adherence in pediatric long-term medical conditions: systematic review and synthesis of qualitative studies of caregivers' views. BMC Pediatr. 2014 Mar 4;14:63.

7. Knafl K, Leeman J, Havill NL, Crandell JL, Sandelowski M. The Contribution of Parent and Family Variables to the Well-Being of Youth With Arthritis. J Fam Nurs. 2015 Nov;21(4):579-616.

8. McDonagh JE, Shaw KL, Prescott J, Smith FJ, Roberts R, Gray NJ. "Sometimes I feel like a pharmacist": identity and medication use among adolescents with juvenile arthritis. Pediatr Rheumatol Online J. 2016 Oct 19;14(1):57.

9. Gomez-Ramirez O, Gibbon M, Berard R, Jurencak R, Green J, Tucker L, et al. A recurring rollercoaster ride: a qualitative study of the emotional experiences of parents of children with juvenile idiopathic arthritis. Pediatr Rheumatol Online J. 2016 Mar 9;14(1):13.

10. Chaney JM, Gamwell KL, Baraldi AN, Ramsey RR, Cushing CC, Mullins AJ, et al. Parent Perceptions of Illness Uncertainty and Child Depressive Symptoms in Juvenile Rheumatic Diseases: Examining Caregiver Demand and Parent Distress as Mediators. J Pediatr Psychol. 2016 Oct;41(9):941-51.

11. Chausset A, Gominon AL, Montmaneix N, Echaubard S, Guillaume-Czitrom S, Cambon B, et al. Why we need a process on breaking news of Juvenile Idiopathic Arthritis: a mixed methods study. Pediatr Rheumatol Online J. 2016 May 21;14(1):31.

12. Yuwen W, Lewis FM, Walker AJ, Ward TM. Struggling in the Dark to Help My Child: Parents' Experience in Caring for a Young Child with Juvenile Idiopathic Arthritis. J Pediatr Nurs. 2017 Nov - Dec;37:e23-e9.
