# Supplementary material for: Development and validation of the CAREGIVERS questionnaire: multi-assessing the impact of juvenile idiopathic arthritis on caregivers
Source: Pediatr Rheumatol Online J. 2020 Jan 14;18:3. doi: 10.1186/s12969-020-0400-z (PMC6961380; doi:10.1186/s12969-020-0400-z)
Supplement: Supplementary file 2 — Additional file 2. Supplementary figure: Overview of search results and review. [file 12969_2020_400_MOESM2_ESM.pptx]

## Slide 1
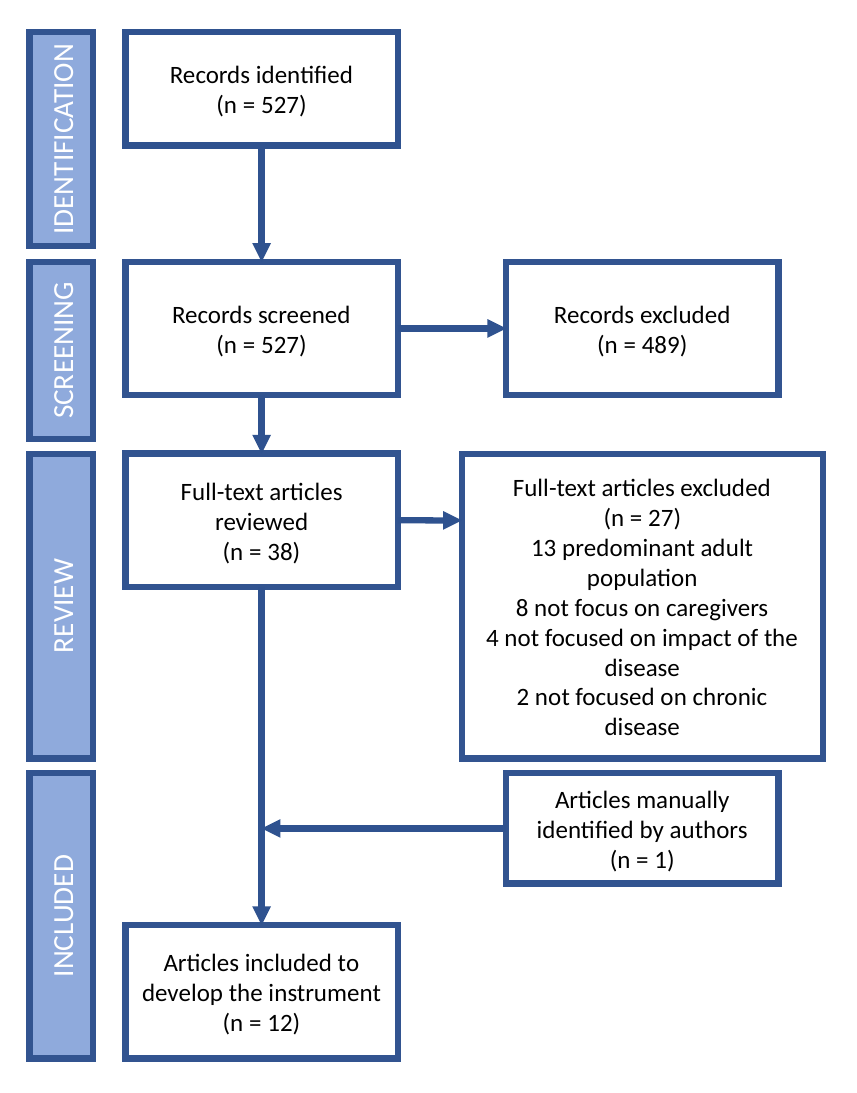

IDENTIFICATION
Records identified
(n = 527)
SCREENING
Records screened
(n = 527)
Records excluded
(n = 489)
REVIEW
Full-text articles reviewed
(n = 38)
Full-text articles excluded
(n = 27)
13 predominant adult population
8 not focus on caregivers
4 not focused on impact of the disease
2 not focused on chronic disease
INCLUDED
Articles manually identified by authors
(n = 1)
Articles included to develop the instrument
(n = 12)
