# Supplementary material for: Development and validation of the CAREGIVERS questionnaire: multi-assessing the impact of juvenile idiopathic arthritis on caregivers
Source: Pediatr Rheumatol Online J. 2020 Jan 14;18:3. doi: 10.1186/s12969-020-0400-z (PMC6961380; doi:10.1186/s12969-020-0400-z)
Supplement: Supplementary file 4 — Additional file 4. CAREGIVERS questionnaire: Impact of Pediatric Rheumatic Diseases on Caregivers Multi-Assessment Questionnaire. [file 12969_2020_400_MOESM4_ESM.doc]

**Supplementary file 4. CAREGIVERS QUESTIONNAIRE: Impact of Pediatric Rheumatic Diseases on Caregivers Multi-Assessment Questionnaire.**

**CAREGIVERS Questionnaire overview**.

| **Dimension** | **Number of items** |
| --- | --- |
| **I. Disease impact** | **6** |
| **II. Social impact** | **3** |
| **III. Economic and working impact**  **IIIA. Financial impact**  **IIIB. Work impact** | **4**  **3** |
| **IV. Family impact** | **5** |
| **V. Impact on caregiver-patient relationship** | **1** |
| **VI. Impact on couple relationship** | **1** |
| **VII. Impact on spirituality / religion / personal beliefs** | **1** |
| **VIII. Impact on social networks** | **4** |
| **Total** | **28** |

**CAREGIVERS QUESTIONNAIRE.**

**This is an illustrative simple translation from the Spanish original version of the instrument.**

1. **EMOTIONAL IMPACT**

**1. How did you feel when you learned that your child / patient had a rheumatic disease?**

a) Sad

b) Angry

c) Guilty

d) With fear

e) I did not accept the reality (Denial)

f) Concerned

g) It did not affect me

h) Relieved

i) Quiet

**2. At this time, how do you feel that your child / patient has a rheumatic disease?**

a) Sad

b) Angry

c) Guilty

d) With fear

e) I do not accept the reality (Denial)

f) Concerned

g) It does not affect me

h) Relieved

i) Quiet

**3. What worries you the most about your child's / patient’s rheumatic disease?**

a) That he / she feels pain.

b) That he / she has difficulty moving (running, walking).

c) Covering the expenses of the treatment.

d) Being able to attend medical appointments.

**4. What do you think the future of your child / patient will be with a rheumatic disease?**

a) With many problems in his / her life

b) With some problems in his / her life

c) No problems in his / her life

**5. Does this cause you anxiety or stress?**

A) Yes

b) No

**6. How do you feel about sharing that your child / patient has a rheumatic disease with other people?**

a) I feel sad

b) I feel ashamed

c) I worry that they make fun of him / her

d) I don't like to share it

e) I feel relieved

f) I feel calm

**II. SOCIAL IMPACT**

**1. Have you changed the way you spend your time since you learned about your child's / patient’s rheumatic disease?**

a) It has changed a lot

b) It has not changed

c) It has changed little

**2. Has your social life changed (walking, traveling, friends, meetings, parties, etc.) since you learned about your child's / patient’s rheumatic disease?**

a) It has changed a lot

b) It has not changed

c) It has changed little

**3. Has your health changed since you learned about your child's / patient’s rheumatic disease?**

a) I neglected my health

b) I got sick

c) It did not change

d) I take more care of my health

**III. ECONOMIC AND LABOR IMPACT**

**III.A ECONOMIC IMPACT**

**1. Has your family economic situation changed since you learned about your child's / patient’s rheumatic disease?**

a) The economic situation worsened

b) The economic situation did not change

c) The economic situation improved

**2. Have you, your partner or someone in your family had to borrow money to treat your child's / patient’s rheumatic disease?**

A) Yes

b) No

**3. Have you stopped buying your child's / patient's medications because of lack of money?**

A) Yes

b) No

**4. Have you received additional support (money, medications, food, etc.) to treat your child's / patient’s rheumatic disease?**

A) Yes

b) No

**III.B LABOR IMPACT**

**1. Have you had problems at work because of attending to your child's / patient’s rheumatic disease?**

a) I lost my job

b) I had to work more

c) I had to get a job

d) I had to miss days at work

e) I have changed jobs

f) I have had no problems at work

g) Does not apply

**2. Has your partner had problems at work because of attending to your child's / patient’s rheumatic disease?**

a) He has lost his job

b) He has had to work more

c) He had to get a job

d) You have had to miss your job

e) He has changed jobs

f) He has had no problems at work

g) Does not apply

**3. Has anyone in your family had problems at work because of attending to your child’s / patient’s rheumatic disease?**

a) Someone has lost their job

b) Someone has had to work more

c) Someone has had to get a job

d) Someone has had to miss work

e) Someone has had to changed jobs

f) No one has had problems at work

g) Does not apply

**IV. FAMILY IMPACT**

**1. Has the way in which you interact with your family changed since you learned about your child's / patient’s rheumatic disease?**

a) It has worsened

b) It has not changed

c) It has improved

**2. Do you need, or have you needed to ask your family for help in order to attend to the rheumatic disease of your child / patient?**

A) Yes

b) No

**3. During the rheumatic illness of your child / patient, who helps you with domestic activities?**

a) Partner

b) Family members

c) Domestic employee

d) Friends

e) No one helps me

**4. How satisfied are you with this help?**

a) Very satisfied

c) Little satisfied

d) Not satisfied

e) Does not apply

**5. Who accompanies you to your child's / patient’s medical appointments?**

a) Partner

b) Family members

c) Domestic employee

d) Friends

e) No one accompanies me

**V. IMPACT ON CAREGIVER-PATIENT RELATIONSHIP**

**1.Has the relationship with your child / patient changed since the diagnosis of his / her rheumatic disease?**

a) We have lost the relationship

b) We have moved away

c) Our relationship has not changed

d) We have approached

**VI. COUPLE RELATIONSHIP IMPACTS**

**1. Has your relationship changed as a result of the rheumatic illness of your child / patient?**

a) Abandonment

b) Divorce / Separation

c) Distance (We distance ourselves as a couple)

d) It has not changed

e) We are closer than before

f) Change partners

g) I have no partner

**VII. IMPACT ON SPIRITUALITY / RELIGION / PERSONAL BELIEFS**

**1. Has your spirituality, religion or personal beliefs changed since the diagnosis of rheumatic disease of your child / patient?**

a) I abandoned my religion / beliefs

b) I feel further away from my religion / beliefs

c) I changed my religion / beliefs

d) I did not change religion / beliefs

e) I have never believed in anything

f) I am closer to religion and my beliefs

**VIII. IMPACT OF SOCIAL NETWORKS**

**1. Have you searched for information about your child's / patient’s rheumatic disease in the internet?**

A) Yes

b) No

c) I am not interested

d) Not applicable (I don't have internet)

**2. How has the information about your child’s / patient’s rheumatic disease in the internet have affected you?**

a) It has caused me anxiety / stress

b) It has confused me

c) It has not helped me

d) It has helped me

e) Does not apply

**3. Have you used social media to communicate with other parents / caregivers of children suffering from the same rheumatic disease as your child / patient?**

A) Yes

b) No

c) Does not apply

**4. How did you feel about communicating with other people through social networks?**

a) It caused me anxiety / stress

b) It confused me

c) It has not helped me

d) It has helped me

e) Does not apply
